# Central retinal artery occlusion combined with posterior ciliary artery occlusion after preoperative embolization of the middle meningeal artery for intracranial meningioma: A case report

**DOI:** 10.1097/MD.0000000000021197

**Published:** 2020-07-10

**Authors:** Kathy Ming Feng, Chang-Min Liang, Shu-I Pao

**Affiliations:** a Department of Ophthalmology; b Graduate Institute of Medical Sciences; c Department of Ophthalmology, School of Medicine, Tri-Service General Hospital, National Defense Medical Center, Taipei, Taiwan, Republic of China.

**Keywords:** central retinal artery occlusion, central retinal artery occlusion, embolization, meningioma, posterior ciliary artery occlusion

## Abstract

**Rationale:**

Preoperative embolization of brain tumors has been widely used to minimize hemorrhaging during surgery, but anastomosis between vessels is sometimes overlooked and complications can occur. Herein we describe a case of rare complications of central retinal artery occlusion (CRAO) and posterior ciliary artery occlusion after embolization of the middle meningeal artery.

**Patient concerns:**

A 48-year-old woman experienced acute, painless loss of vision in her left eye during embolization of the middle meningeal artery for meningioma.

**Diagnosis:**

The patient was diagnosed with CRAO and posterior ciliary artery occlusion based on indirect ophthalmoscopy, optical computed tomography of the macula, and fundus angiography.

**Interventions:**

Ocular massage, oral acetazolamide, and topical brimonidine eyedrops were administered.

**Outcomes:**

Visual acuity decreased from hand motion to no light perception within 2 months. Optic disc atrophy with retinal thinning was evident after 2 to 4 months.

**Lessons:**

The blood supply and any collateral vessels of the ophthalmic artery should be vigilantly scrutinized to prevent complications during embolization of the middle meningeal artery that may lead to a poor visual outcome.

## 1 Introduction

Central retinal artery occlusion (CRAO) after preoperative embolization of the middle meningeal artery for meningioma is a complication that can have devastating effects on the eye. The aim of preoperative embolization of meningioma is to minimize intraoperative hemorrhaging, thus affording surgeons a better surgical view and reducing surgery time.^[1]^ Unintentional embolization of retinal arteries is rare, but it has been reported in conjunction with embolization of the external carotid artery.^[2]^ Herein we report a case of both CRAO and posterior ciliary artery occlusion after preoperative embolization of the middle meningeal artery for meningioma. This case report was approved by the ethics committee of the Tri-Service General Hospital in Taiwan (TSGH IRB No. 2-108-05-140). Written informed consent was obtained from the patient for the publication of this case report and accompanying images.

## 2 Case presentation

**Figure 1:**
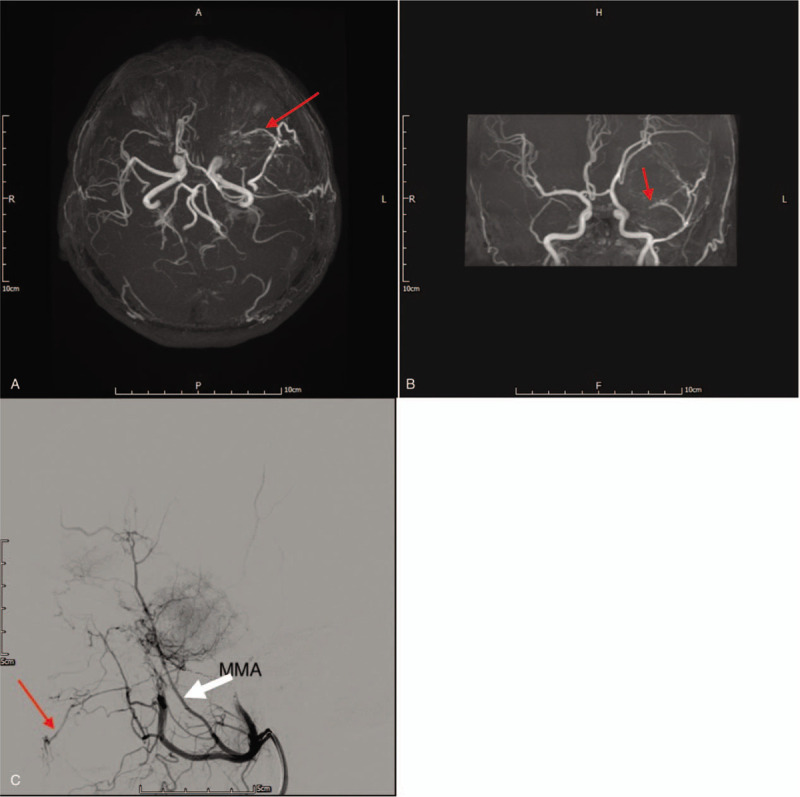
Brain magnetic resonance angiography (A–B) and external carotid angiography (C). (A–C) Red arrows indicate a branch to the left orbit from the middle meningeal artery (MMA). White arrow indicates the left middle meningeal artery.

A 48-year-old woman with hypertension had experienced difficulty naming objects and communicating with others for 2 months. Her ocular history was unremarkable but magnetic resonance imaging revealed a large extra-axial tumor mass (7.5 × 6.0 cm) in the left frontal-temporal region. Magnetic resonance angiography revealed that the tumor was mainly supplied by the middle meningeal artery, and a branch artery flowed into the left orbit (Fig. 1). A total of 9 fiber coils and 400 μm microspheres were used in the middle meningeal artery to occlude the orifices of supplying branches. Blurred vision in the left eye was reported by the patient however, and an ophthalmologist was immediately consulted.

**Figure 2:**
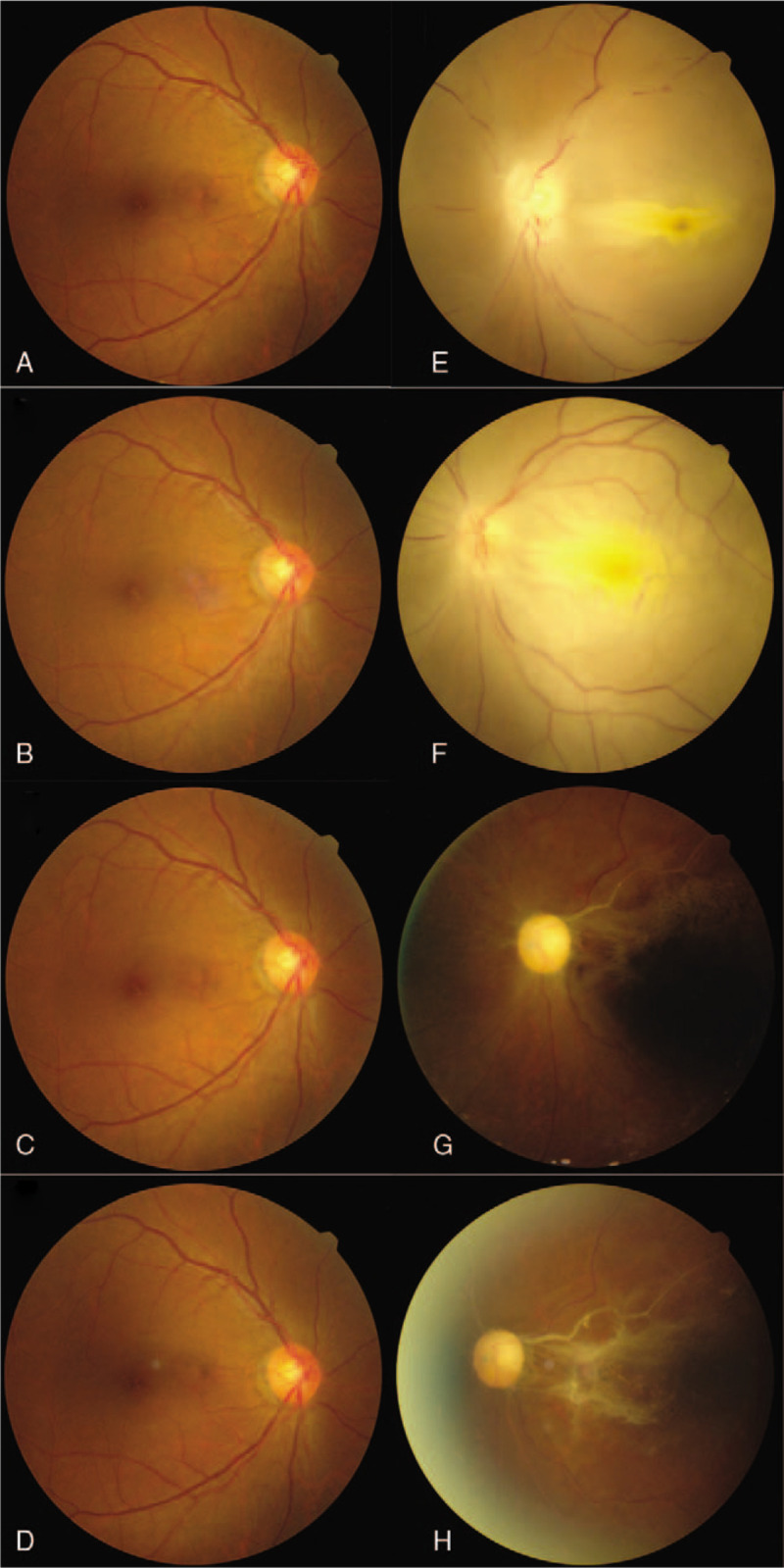
Fundus examination of both eyes immediately after preoperative embolization, 1 week after embolization, 2 months after embolization, and 4 months after embolization. (A–D) The right eye exhibited normal fundus appearance. (E) After embolization the left eye exhibited retinal whitening with milky-white edema at the papillomacular bundle and disc swelling. (F) After 1 week, the left eye showed persistent disc swelling and diffuse milky-white edema at macula. (G) After 2 months there was a pale disc with peripapillary sheathing of retinal vasculature in the left eye. (H) After 4 months there was optic disc atrophy with diffuse fibrovascular membrane overlying the macular area and sheathing of retinal vessels in the left eye.

**Figure 3:**
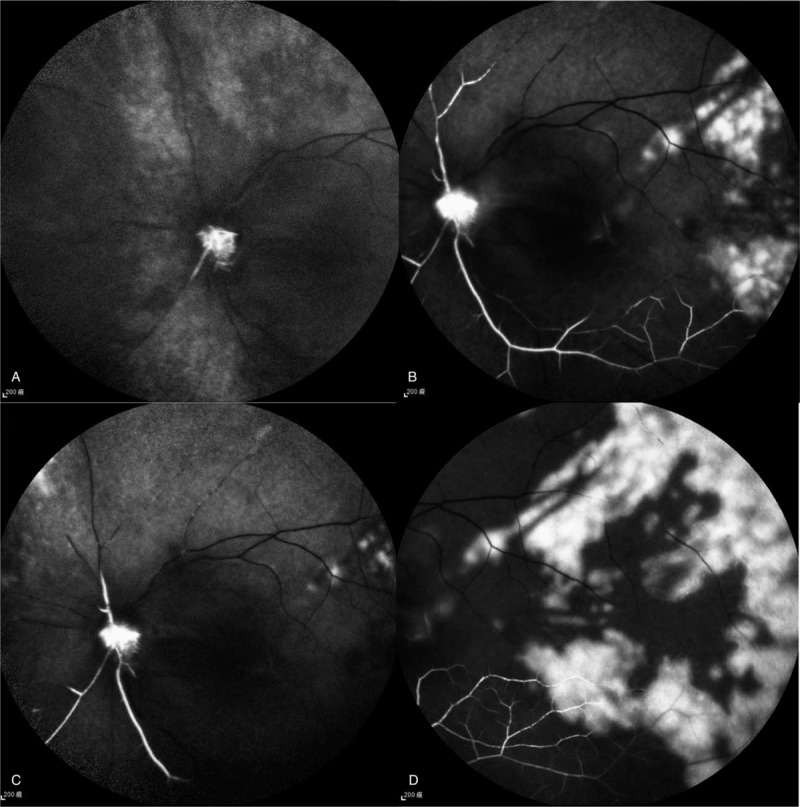
(A) Delayed perfusion at the artery phase with disc leakage and a few patches of non-choroidal filling. (B–E) Late phase: disc leakage with triangular hyperfluorescent lesions and non-filling arteries are depicted.

**Figure 4:**
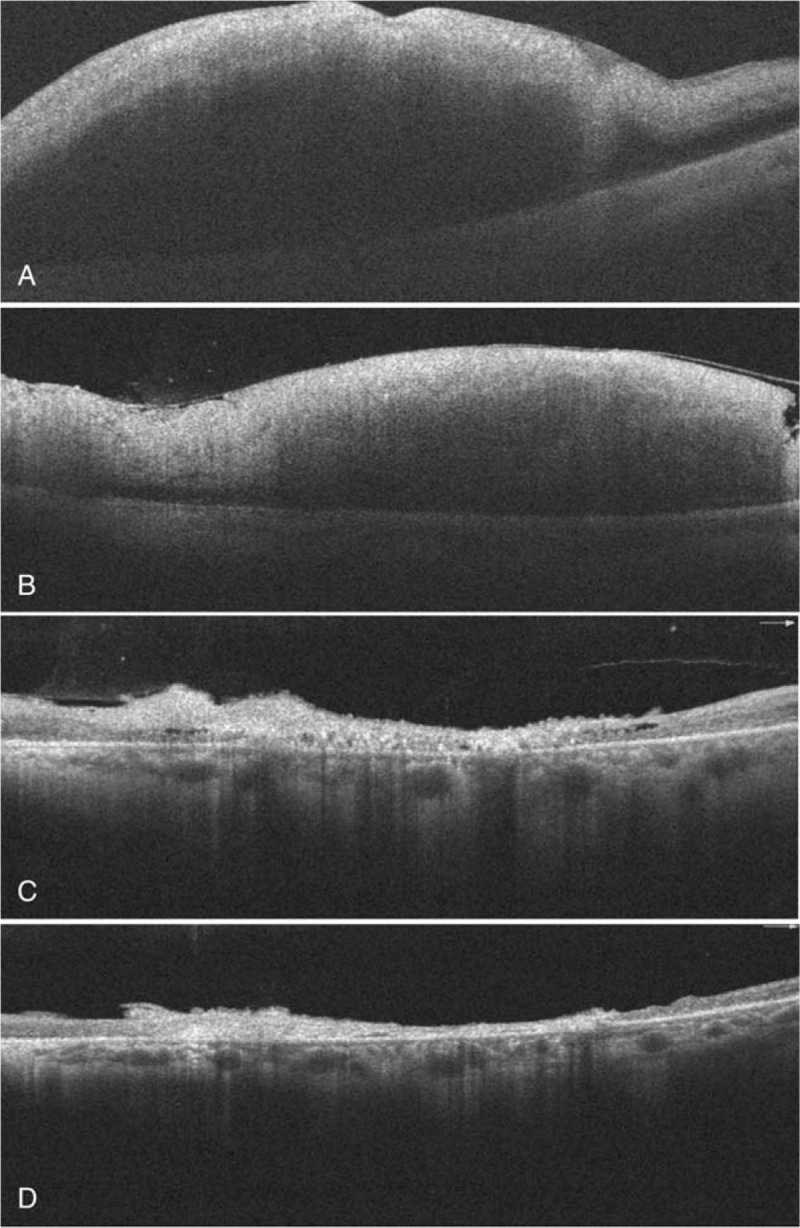
Spectral domain optical coherence tomography of the left eye. (A) After embolization, there was diffuse swelling of all retinal layers. (B) At the end of week 1 diffuse retinal swelling persisted, with less subretinal fluid. (C) Two months after the embolization, the swelling had improved and there was hyperreflectivity and thinning of retinal layers. (D) Four months after the embolization, there was still hyperreflectivity and diffuse thinning of all retinal layers.

**Figure 5:**
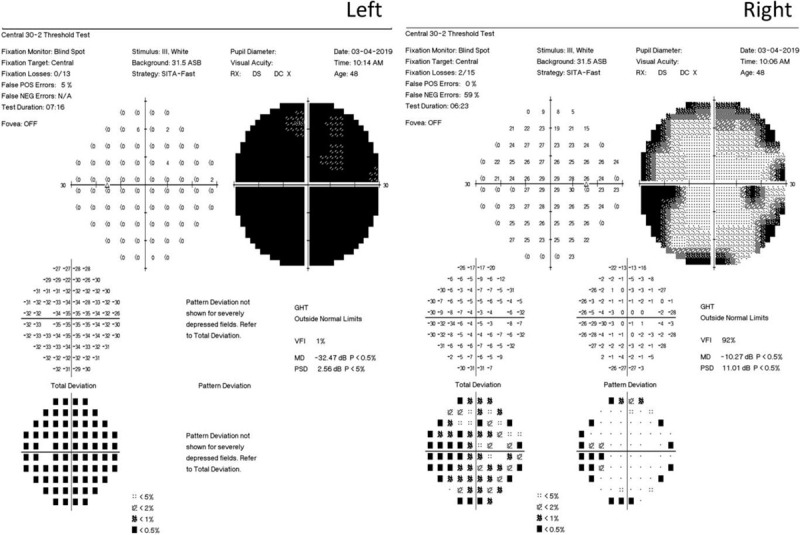
Visual fields of both eyes as determined via the 30–2 hemifield test. Complete scotoma was evident in the left eye, and a constricted field of vision was evident in the right eye.

Ophthalmological examination revealed visual acuity of hand motion in the left eye and 20/20 in the right eye. Intraocular pressure and the anterior segments were normal in both eyes, with the exception of prominent relative papillary afferent defect in the left eye. Fundus examination revealed diffuse retinal whitening and edema, milky-white edema at the papillomacular bundle, and disc swelling in the left eye (Fig. 2), compatible with a diagnosis of central retinal artery occlusion. Immediate ocular massage, oral acetazolamide, and topical brimonidine drops were administered. Fluorescein angiography revealed leakage in the region of the optic disc in the early phase, with several choroidal nonperfusion areas, non-arterial filling, and several triangular hyperfluorescent patches in the late phase (Fig. 3). Optical computed tomography (OCT) revealed retinal edema of the left eye (Fig. 4). Her visual field parameters included total scotoma in the left eye and peripheral loss in the right eye (Fig. 5). Post-embolization the tumor exhibited significantly reduced contrast. The patient underwent brain tumor removal surgery 1 week later and meningioma grade I was confirmed by a pathologist. After 2 months visual acuity in the left eye was no light perception, and fundus examination revealed optic disc atrophy with peripapillary sheathing of the arteriolar vasculature (Fig. 2). OCT revealed irregular macular edema with hyperreflectivity of the thickened retina (Fig. 4). Four months later there was no improvement in visual acuity in the left eye, and fundus examination revealed diffuse fibrovascular membrane with inner retinal atrophy and an absence of identifiable retinal layers on OCT (Fig. 2).

## 3 Discussion

CRAO following preoperative embolization of the feeder artery for meningioma is a known potential complication and constitutes an ophthalmic emergency.^[3,4]^ Prompt treatment may reduce the risk of permanent visual impairment, but the majority of patients experience reduced visual acuity thereafter. In the present case the patient developed an acute onset of CRAO combined with posterior ciliary artery occlusion after embolization of the middle meningeal artery for meningioma.

Meningiomas are dural-based tumors that arise from arachnoid cells. They have a multidirectional blood supply but are mainly supplied by middle meningeal, accessory meningeal, or ascending pharyngeal arteries, or the occipital branch of the external carotid artery.^[5]^ Preoperative embolization of the feeder artery aids surgeons by reducing bleeding during surgical resection, which can minimize blood loss and subsequently reduce operating time.^[1]^ It is often performed using coils and glues. In the present case 400-μm Embozene and 9 fiber coils were used. Embozene consists of soft, deformable embolizing microspheres that contain a hydrogel core of polymethylmethacrylate and can travel into deep structures of a tumor.^[6]^ While many consider embolization a safe technique, complications can occur; including cranial nerve palsy, tissue necrosis, intracerebral hemorrhage, and contrast-induced nephropathy. Accidental embolization to the retinal arteries is rare, but it has been documented in a few case reports.^[2–4]^ The ophthalmic artery typically branches off from the internal carotid artery, but variants may arise from the middle meningeal artery, or anastomosis may exist between them.^[7]^ Potential anastomosis between the middle meningeal artery and ophthalmic artery via collateral vessels complicates the embolization procedure. The collateral vessels may occur via recurrent meningeal or lacrimal arteries or other vessels.^[6]^ In the current patient magnetic resonance angiography depicted the middle meningeal artery supplying the tumor, and the patient had a branch of the artery flowing into the left orbit, suggesting that the ophthalmic artery may also have been supplied by the middle meningeal artery. Unfortunately it was not possible to perform internal carotid artery angiography to determine whether it also supplied the ophthalmic artery. The patient underwent embolization through the external carotid artery, and reduced vascularity of the tumor was evident post-embolization. The onset of acute painless loss of vision in the left eye then occurred however, and CRAO combined with posterior ciliary artery occlusion were subsequently confirmed via examination. It was postulated that the aforementioned microspheres may have migrated through the branch of the middle meningeal artery to the ophthalmic artery, and subsequently blocked the central retinal artery and posterior ciliary artery.

CRAO is a potentially devastating ophthalmic emergency that can lead to permanent visual dysfunction. Its incidence rate is approximately 1 or 2 per 100,000 people^[8,9]^ and it is usually associated with arterial hypertension, diabetes mellitus, carotid artery disease, cerebrovascular accident, and/or ischemic heart disease.^[10]^ Although occurrences are rare various procedures may be associated with embolic complications involving the retinal arteries, such as interventional radiology, retrobulbar anesthesia, and endoscopic sinus surgery.^[11,12]^ The current patient experienced permanent CRAO involving both the central retinal artery and the posterior ciliary artery. The central retinal artery provides circulation to the inner retina, whereas the posterior ciliary artery supplies the outer retina, choroid, and optic nerve head.^[13,14]^ In the present case early fluorescence angiography depicted an absence of choroidal fluorescence, and in the late phase there were triangular hyperfluorescent patches, confirming choroidal ischemia. Due to poor blood supply to the choroid, optic nerve head, and retina, fundus examination after 2 months revealed optic disc atrophy and thinning of the whole retina and choroid. In a previous study of 260 eyes in 244 patients with CRAO, 74.2% exhibited visual acuity of counting fingers or worse at initial presentation and in 90% of those eyes there was no subsequent improvement.^[15]^ The current patient's visual acuity deteriorated from hand motion to no light perception within 2 months.

Treatment for CRAO is currently challenging because there is no well-established treatment protocol. In current conventional therapy intraocular pressure lowering medication, anterior chamber paracentesis, and ocular massage are used. Some clinicians advise the use of hyperbaric oxygen therapy to facilitate an ischemic inner retina^[16]^; nevertheless, the frequency and duration of hyperbaric oxygen therapy remain controversial.

In summary, with the increasing use of preoperative embolization of meningioma to reduce intraoperative hemorrhaging, clinicians should be aware of the arteries that branch into the ophthalmic artery. They should also have a predetermined management plan devised, ready to be instigated in the event that the patient complains of sudden loss of vision. In the present case occlusion of the retinal artery after preoperative embolization of meningioma led to severe loss of vision.

## Author contributions

**Conceptualization:** Kathy Ming Feng, Chang-Min Liang, Shu-I Pao.

**Data curation:** Kathy Ming Feng, Chang-Min Liang, Shu-I Pao.

**Formal analysis:** Kathy Ming Feng, Shu-I Pao.

**Funding acquisition:** Chang-Min Liang.

**Investigation:** Kathy Ming Feng.

**Methodology:** Kathy Ming Feng.

**Project administration:** Kathy Ming Feng, Shu-I Pao.

**Resources:** Kathy Ming Feng, Shu-I Pao.

**Supervision:** Chang-Min Liang, Shu-I Pao.

**Validation:** Kathy Ming Feng.

**Visualization:** Kathy Ming Feng.

**Writing – original draft:** Kathy Ming Feng.

**Writing – review & editing:** Shu-I Pao, Chang-Min Liang.
